# MRI‐based radiomics models for early predicting pathological response to neoadjuvant chemotherapy in triple‐negative breast cancer: A systematic review and meta‐analysis

**DOI:** 10.1002/acm2.70296

**Published:** 2025-10-15

**Authors:** Jupeng Zhang, Qi Wu, Peng Lei, Xiqi Zhu, Baosheng Li

**Affiliations:** ^1^ Department of Radiology Affiliated Hospital of Youjiang Medical University for Nationalities Baise China; ^2^ School of Testing Affiliated Hospital of Youjiang Medical University for Nationalities Baise China; ^3^ Life Science and Clinical Medicine Research Center Affiliated Hospital of Youjiang Medical University for Nationalities Baise China

**Keywords:** MRI, neoadjuvant chemotherapy, radiomics, triple‐negative breast cancer

## Abstract

**Objective:**

This meta‐analysis evaluates the accuracy of MRI‐based radiomics in predicting pathological complete response (pCR) to neoadjuvant chemotherapy (NAC) in triple‐negative breast cancer (TNBC) patients.

**Methods:**

A systematic search of PubMed, Cochrane Library, Embase, Scopus, and Web of Science was conducted up to September 2024. Ten studies meeting inclusion criteria were assessed for methodological quality using the Radiomics Quality Score (RQS) and QUADAS‐2 tools. Pooled diagnostic performance metrics, including AUC, sensitivity, and specificity, were calculated using a fixed‐effects model.

**Results:**

The fixed‐effects model yielded a pooled AUC of 0.83 (95% CI: 0.79–0.86), with sensitivity of 0.80 (95% CI: 0.68–0.88) and specificity of 0.85 (95% CI: 0.76–0.91). Support Vector Machine (SVM) and Light Gradient Boosting Machine (LightGBM) classifiers demonstrated the highest diagnostic efficacy (AUC = 0.86). Heterogeneity was low (*I* ^2^ = 32%), supporting the use of a fixed‐effects approach.

**Conclusion:**

MRI‐based radiomics exhibits strong and consistent predictive performance for pCR in TNBC patients undergoing NAC, supporting its potential as a non‐invasive tool for early treatment response assessment. Further standardization and prospective validation are needed for clinical implementation.

## 1 INTRODUCTION

Breast cancer (BC) remains the most prevalent malignancy and second leading cause of cancer mortality among women worldwide.^1^ Triple‐negative breast cancer (TNBC) accounts for 15%–20% of all BC cases.^2^, ^3^ This aggressive subtype exhibits particularly poor prognosis due to its high metastatic potential and limited treatment options.^3^ For locally advanced TNBC, neoadjuvant chemotherapy (NAC) has become standard therapy, with pathological complete response (pCR) serving as a critical prognostic indicator.^4^, ^5^, ^6^ Achieving pCR, defined as eradication of invasive tumor in both breast and axillary lymph nodes, correlates significantly with improved disease‐free and overall survival.^6^, ^7^ Current NAC regimens yield pCR rates of only 30–40%, while exposing patients to substantial toxicity risks including hematologic, hepatic, and cardiac complications.^8^, ^9^, ^10^ These clinical challenges underscore the urgent need for reliable early response assessment methods to optimize treatment strategies.

Current clinical imaging modalities for NAC response assessment—including ultrasound, CT, mammography, and MRI^11^, ^12^, ^13^ face limitations in accurately predicting pCR. While RECIST criteria classify tumor responses based on volumetric changes,^12^ these often poorly correlate with pathological outcomes. MRI has emerged as the most reliable modality, demonstrating superior pCR prediction capabilities through precise morphological assessment in landmark trials like ACRIN 6657/I‐SPY.^13^, ^14^, ^15^, ^16^

Radiomics represents a transformative approach in quantitative imaging analysis, extracting high‐dimensional data to enhance predictive accuracy.^17^, ^18^ By integrating multiparametric imaging features with clinical and molecular data, radiomics enables more precise treatment response evaluation.^19^ Initial applications in TNBC have shown promising results, with reported AUC values of 0.8–0.89 for pCR prediction,^20^, ^21^ suggesting its potential for personalized therapeutic decision‐making.

Despite growing interest in MRI‐based radiomics for TNBC management, the diagnostic performance remains inconsistent across studies. Inconsistent diagnostic performance may result from differences in MRI acquisition protocols, heterogeneity in segmentation methods, and differences in feature extraction platforms, and so forth.^22^, ^23^ This systematic review and meta‐analysis therefore aims to comprehensively evaluate the methodological quality and predictive value of radiomics approaches for pCR assessment in TNBC patients receiving NAC.

## 2 METHODS

### 2.1 General guidelines

This systematic review followed PRISMA‐DTA guidelines^24^ and was prospectively registered in XXX (XXXX). Ethical approval was waived as no human subjects were involved.

### 2.2 Database search and identification of eligible manuscripts

Two investigators (ZJ, WQ) independently searched PubMed, Cochrane, Embase, Scopus, and Web of Science from inception through September 2024. Discrepancies were resolved by a third reviewer. The complete search strategy is provided in Supplementary information. In addition, four key methodological components were required in all included studies: (a) MRI acquisition using a standardized protocol (1.5T or 3.0T system), (b) tumor segmentation (manual or semi‐automated methods), (c) radiomics feature extraction using a validated platform (PyRadiomics or MATLAB), and (d) development of a machine‐learning algorithm using a Predictive Modeling.

### 2.3 Inclusion and exclusion criteria

The inclusion criteria for studies were: (1) TNBC patients undergoing neoadjuvant therapy; (2) publications in English; (3) pathologic evaluation of surgical specimens as the reference standard; and (4) Reported AUC values with corresponding 95% CI, SD, or SE. and (5) studies predicting neoadjuvant chemotherapy based on radiomics modeling. Exclusion criteria were as follows: (1) patients with other types of concurrent primary cancers; (2) patients with distal metastases from breast cancer; and (3) article type of review, abstract, case report, or editorial material.

### 2.4 Data extraction and management

Data were extracted independently by two reviewers (ZJ and WQ) using a predefined form, including items such as first author, publication year, country, study type, MR equipment, field strength, cross‐validation or test set, patient disease duration, NAC regimen, NAC definition, feature extraction software, algorithm architecture, and radiomics used. Disagreements were resolved through discussion to reach consensus.

### 2.5 Quality assessment

Two independent reviewers (ZJ and WQ) evaluated study quality using QUADAS‐2^25^ and the Radiomics Quality Score (RQS).^18^ The RQS, a validated framework for radiomics study assessment developed by Lambin et al.,^18^ quantifies technical reproducibility and clinical validation completeness.

### 2.6 Statistical analysis

In the main pooled analyses AUC values were summarized using the generalized inverse variance method, that is, pooled effect sizes and standard errors were extracted from the original papers and, if missing, effect sizes and standard errors were calculated. The results of the studies were also weighted using the inverse variance method, giving greater weight to those with higher precision (narrower confidence intervals). This method provides a more precise estimate of the larger effects in the combined analysis. The analysis used R^26^ of metafor^27^ package for effect size combinations and the metadisc package to generate forest plots. Fixed‐effects models were selected when heterogeneity was low (< 50%). For studies reporting multiple models, only the highest AUC was included. results from external validation sets were prioritized if available; otherwise, results from internal validation sets or training sets were used.

For the secondary analyses, that is, sensitivity, specificity, AUC, and Hierarchical Receptor Operating Characteristic Curve (HSROC) parameters for six of the studies, complete four‐cell tabular data were required for each study. These metrics were analyzed using the bivariate random‐effects model of Reitsma^28^ et al. in the mada^29^ package, which takes into account the inherent negative correlation between sensitivity and specificity across studies. dmetatools package^30^ was used to compute bootstrap confidence intervals for all diagnostic accuracy metrics, including positive/negative likelihood ratios (PLR/NLR) and diagnostic odds ratios (DOR). The inconsistency index (*I* ^2^) was used to assess the percentage of total variation due to heterogeneity across studies. If the *I* ^2^ value is greater than 50%, it indicates significant heterogeneity; if the *I* ^2^ value is less than 50%, it indicates less heterogeneity.

Subgroup analyses and meta‐regression analyses were assessed using the metadisc software package for *R* and stata 16.0, respectively. For subgroup analysis we analyze the diagnostic efficacy of five classifiers of Logistic Regression (LR), Support Vector Machine (SVM), LightGBM, Not Reported (NR), and K‐Nearest Neighbor (K‐NN). As for meta‐regression we constructed a total of five regression models containing models for covariates such as country, study type, field strength, validation (cross‐validation or test set), and pCR definition. The country covariate indicates the country or region of data source. And the study type covariate indicates whether the included study was prospective or regression. The field strength covariate reflected whether the device was 3.0 T or 1.5 T. The validation covariate reflected whether the included studies were cross‐validated or assessed by a training group on the efficacy of the constructed model. And the definition covariate of pCR mainly refers to the specific effect after neoadjuvant chemotherapy in the included studies. Covariates were statistically significant at *p* < 0.05.

Publication bias was assessed using funnel plots (Deek^,^s)^31^ and Egger's test using R's metafor software package to ensure whether there was a risk of publication bias. The method of Deek^,^s et al. ^32^ is considered to be the most appropriate method for assessing publication bias in diagnostic test accuracy studies. *p* < 0.05 may indicate the presence of publication bias.

## 3 RESULTS

### 3.1 Identification of studies

**FIGURE 1:**
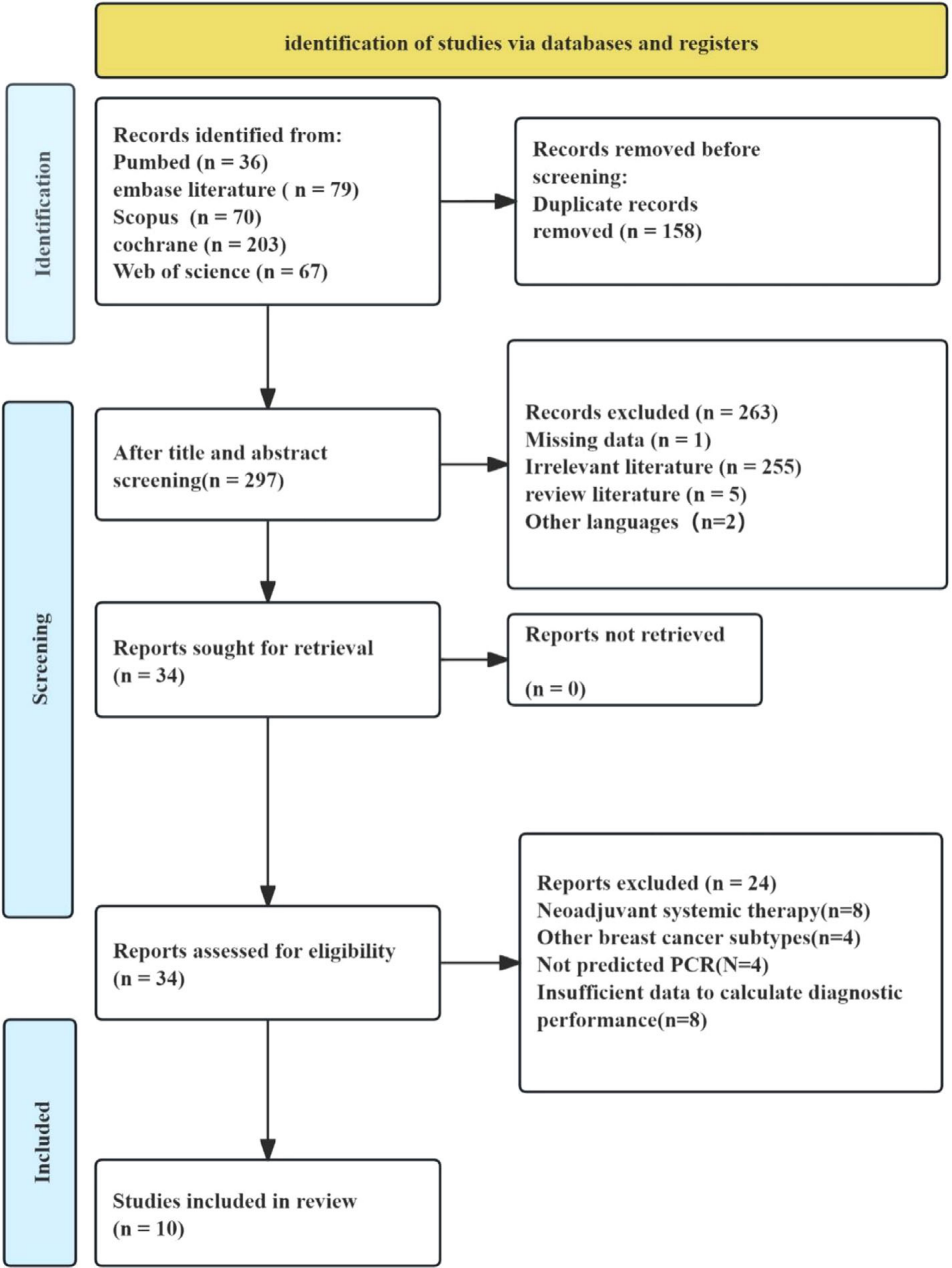
Preferred reporting items for systematic reviews and meta‐analyses (PRISMA) flow diagram illustrating the number of studies screened and excluded.

From the 455 records initially identified, 10 studies^33^, ^34^, ^35^, ^36^, ^37^, ^38^, ^39^, ^40^, ^41^, ^42^ met all inclusion criteria after double deletion and two‐stage screening. The study screening process is shown in the PRISMA flowchart in Figure 1.

### 3.2 Characteristics of included studies

We included 10 studies, with basic characteristics shown in Table 1 and baseline characteristics shown in Table 2. In terms of geographic level, six were from China,^33^, ^35^, ^36^, ^39^, ^40^, ^42^ one from the Netherlands,^41^ one from Korea,^38^ one from France,^37^ and one from USA.^34^ Eight studies were retrospective,^33^, ^34^, ^36^, ^38^, ^39^, ^40^, ^41^, ^42^ and two were prospective.^35^, ^37^ In terms of magnetic resonance imaging protocols, all studies used DCE‐MRI as the core imaging modality, and the auxiliary sequences included T1WI. Regarding coil configurations, six studies^33^, ^34^, ^37^, ^39^, ^41^, ^42^ did not report the number of channels, two studies^35^, ^36^ used 16‐channel breast coils, one study^40^ employed an 8‐channel coil, and one study^38^ utilized a 4‐channel coil.

**TABLE 1:** Patient and study characteristics.

Authors	Year	Country	Duration of the patie	Study design	MR scanner	magnetic field strength(T)	Slice thickness	Breast coil	Check Sequence	MRI examination	Cross‐validation or test set	Neoadjuvant therapy programs	pCR Definition	Non‐pCR definitions
Marco Caballo et al.	2022	Netherlands	2000‐2014	R	GE/Siemens	Both 1.5 and 3.0	NR	NR	T1WI/DCE	Biopsy and pre‐NAC	leave‐one‐out cross validation	NR	No disease (invasive or in situ) in the breast or axillary lymph nodes	Defined as obtaining only a partial response (or no response), or remaining ductal or lobular carcinoma in situ
Jiamin Guo et al.	2024	China	2012‐2021	R	NR	3.0	4mm	8‐ channel coil	T1WI/T2WI/DCE/DWI	Biopsy and pre‐NAC	Five‐fold cross validation	Anthracyclines and paclitaxel are the mainstay, and platinum‐containing chemotherapy is used in some high‐risk patients.	No invasive carcinoma residue was seen in the breast or axillary lymph nodes	Patients with residual lesions in the breast or axilla or both were considered not to have achieved pCR.
Bingqing Xia et al.	2021	China	2011‐2017	R	AURORA	1.5	1.5mm	NR	T1WI/DCE	Biopsy and pre‐NAC	100/50	1. sequential/combined anthracycline neoadjuvant chemotherapy with paclitaxel. 2. with or without platinum	Defined as ypT0/is and ypN0, indicating no residual infiltration	NR
YuHong Huang et al.	2023	China	2015‐2021	R	NR	Both 1.5 and 3.0	NR	16‐channel breast coil	T1WI/T2WI/DCE/DWI	Biopsy and pre‐NAC	409/343,170,340	Based on paclitaxel or paclitaxel in combination with anthracyclines	Defined as ypT0/is/ypN0.	Residual invasive breast cancer or axillary lymph node metastasis
Xue Li et al.	2024	China	2000‐2014	R	NR	Both 1.5 and 3.0	NR	NR	T1WI/DCE	Biopsy and pre‐NAC	217/64	NR	No residual invasive cancer (residual ductal carcinoma in situ acceptable; ypT0/isN0).	NR
Ying Zhang et al.	2022	China	2018‐2019	P	GE/Siemens	Both 1.5 and 3.0	3mm	16‐channel breast coil	T1WI/T2WI/DCE	Biopsy and pre‐NAC	Five‐fold cross validation	Paclitaxel‐based, anthracycline‐based or paclitaxel and anthracyclines	No invasive carcinoma in the breast or associated axillary lymph nodes (ypT0/is ypN0).	NR
Hyo‐jae Lee et al.	2024	South Korea	2015‐2022	R	Siemens	3.0	2mm	4‐channel breast coil	T1WI/T2WI/DCE/DWI	Biopsy and pre‐NAC	Five‐fold cross validation	1. paclitaxel/docetaxel + epirubicin 2. adriamycin + cyclophosphamide, paclitaxel + carboplatin 3. anthracycline and cyclophosphamide combined with programmed cell death protein 1‐targeted monoclonal antibody 4. Fluorouracil + Adriamycin + Cyclophosphamide	No residual invasive tumor in the breast or lymph nodes after surgery	NR
Toulsie Ramtohul et al.	2024	France	2021‐2023	P	NR	NR	1.2mm	NR	T1WI/DCE	Biopsy and pre‐NAC	112/83	Pembrolizumab plus paclitaxel plus carboplatin followed by pembrolizumab plus adriamycin or epothilone plus cyclophosphamide	NR	NR
Tianwen Xie et al.	2022	China	2016‐2021	R	GE/Siemens	Both 1.5 and 3.0	2mm	NR	T1WI/DCE	Biopsy and pre‐NAC	93/113/76	Epirubicin/cyclophosphamide + doxorubicin, doxorubicin/carboplatin	Defined as no infiltrative or non‐infiltrative residue in breast or axillary lymph nodes (ypT0 ypN0)	NR
Sadia Choudhery et al.	2022	USA	2009‐2016	R	GE	1.5	2.6mm	NR	T1WI/T2WI/DCE/DWI	Biopsy and pre‐NAC	NR	NR	No residual invasive tumor in the surgically removed breast and/or ipsilateral axillary lymph nodes	NR

Abbreviations: NR, not reported; R, A retrospective study; P, A prospective study; GE, general electric; MR, magnetic resonance; pCR, pathologic complete response; DWI, diffusion weighted imaging; DCE, Dynamic contrast‐enhanced; T1WI, T1‐weighted images; T2WI, T2‐weighted images.

**TABLE 2:** Baseline characteristics.

Authors	Number of pCRs	No‐pCR number	Type of features	AUC(95%CI)	Feature extraction	Model algorithms	Radiomic used
Marco Caballo et al.	19	53	1. post‐contrast texture; 2. time‐dependent texture; 3. pseudo‐four‐dimensional textures; 4. enhanced kinetic heterogeneity; 5. tumor morphology (volume, surface area, solidity, equivalent diameter, sphericity, surface area to volume ratio)	0.803(0.689‐0.897)	MATLAB	LR	ML
Jiamin Guo et al.	34	87	1. Shape radiomics characterization 2. first‐order statistical radiomics features 3. high‐level texture radiomics features	0.827(0.747‐0.889)	UAI Research platform/PyRadiomics	1. LDA2. RF3. MLP 4. NBB 5. SVM 6. LR	ML
Bingqing Xia et al.	9	41	1. first‐order statistical radiomics features 2. Higher‐order texture radiomics features	0.852(0.773–0.932)	3D Slicer software/ PyRadiomics	1.LDA 2. LR 3. NB 4. RF 5. SVM	ML
YuHong Huang et al.	66	176	1. radiomics features (a. Shape based features b. First order features c. GLDM features d. GLSZM features e. NGTDM features f. GLRLM features g. GLCM features) 2. deep learning features	0.901(0.755–1)	3D Slicer software/Pyradiomics	1.logistic regression 2. random forest 3. XGBoost 4. SVM 5. MultiLayer Perception (MLP) neural network	ML
Xue Li et al.	23	65	1. size 2. Shape 3. Texture 4. Enhancement	0.836(0.708‐0.965)	NR	1.SVM 2. RF 3. DT 4.KNN 5.XGBoost	ML
Ying Zhang et al.	28	84	1. First order features 2. Wavelet features 3. Texture features (a. GLDM features, b. GLCM features, c. GLSZM features, d. GLRLM features, e. NGTDM features).	0.87(0.73‐0.91)	3D Slicer software/PyRadiomics	LightGBM	ML
Hyo‐jae Lee et al.	32	97	1. First order features 2. GLCM features 3. GLDM features 4. GLRLM features 5. GLSZM features 6. NGTDM features	0.802(0.699‐0.905)	3D‐slicer/Pyradiomics	NR	ML
Toulsie Ramtohul et al.	54	29	NR	0.86(0.78, 0.94)	3D Slicer /PyRadiomics	NR	NR
Tianwen Xie et al.	22	54	1. Radiomics features 2. shape features 3. First‐order statistical features 4. Texture features	0.65(0.52, 0.78)	ITK‐SNAP/PyRadiomics	K‐NN	ML
Sadia Choudhery et al.	27	47	1. Morphological features (tumor volume, longest axial and volumetric diameters, and sphericity) 2. Textural features including signal intensity (minimum, median, maximum, mean, and standard deviation), entropy, skewness, and kurtosis	0.73(0.61‐0.86)	MATLAB	LR	ML

Abbreviations: AUC, area under the curve; DT, decision tree; XGBoost, eXtreme Gradient Boosting; GLCM, gray level co‐occurrence matrix; GLDM, Gray gray level dependence matrix; GLRLM, gray level run length matrix; GLSZM, gray level size zone matrix; K‐NN, K‐nearest neighbor; LDA, linear discriminant analysis; LightGBM, light gradient boosting machine; LR, logistic regression; ML, machine learning; MLP, multi‐layer perceptron; NB, naive bayes; NBB, naive bayes classifier; NGTDM, neighborhood gray‐tone difference matrix; NR, not reported; RF, random forest; SVM, super vector machine.

In the clinical setting, platinum‐containing regimens are better predictors than other regimens. Although 90% of the studies used a standardized pCR definition (ypT0/is ypN0), validation strategies varied between training‐test segmentation and cross‐validation. This comprehensive analysis revealed two important findings: first, the widespread use of DCE‐MRI for the examination of breast patients; and second, the observed heterogeneity in other technical parameters underscores the need for standardized protocols, which may limit their generalizability. These results both highlight the consistency of the core imaging methods and point to the challenges that remain in harmonizing protocols.

### 3.3 Risk of bias assessment

All 10 studies underwent RQS evaluation, demonstrating excellent inter‐reviewer agreement (ICC = 0.96, 95%CI:0.86‐0.99) (Figure S1). Studies exhibited variable quality, with two scoring highest^36^, ^37^ and two lowest.^34^, ^42^ Common limitations included absent cost‐effectiveness analyses and multi‐scanner validation. Strengths were observed in image protocol quality, feature selection methods, and discrimination statistics. Only one prospective trial was registered,^37^ with remaining studies being retrospective (Table S1).

**FIGURE 2:**
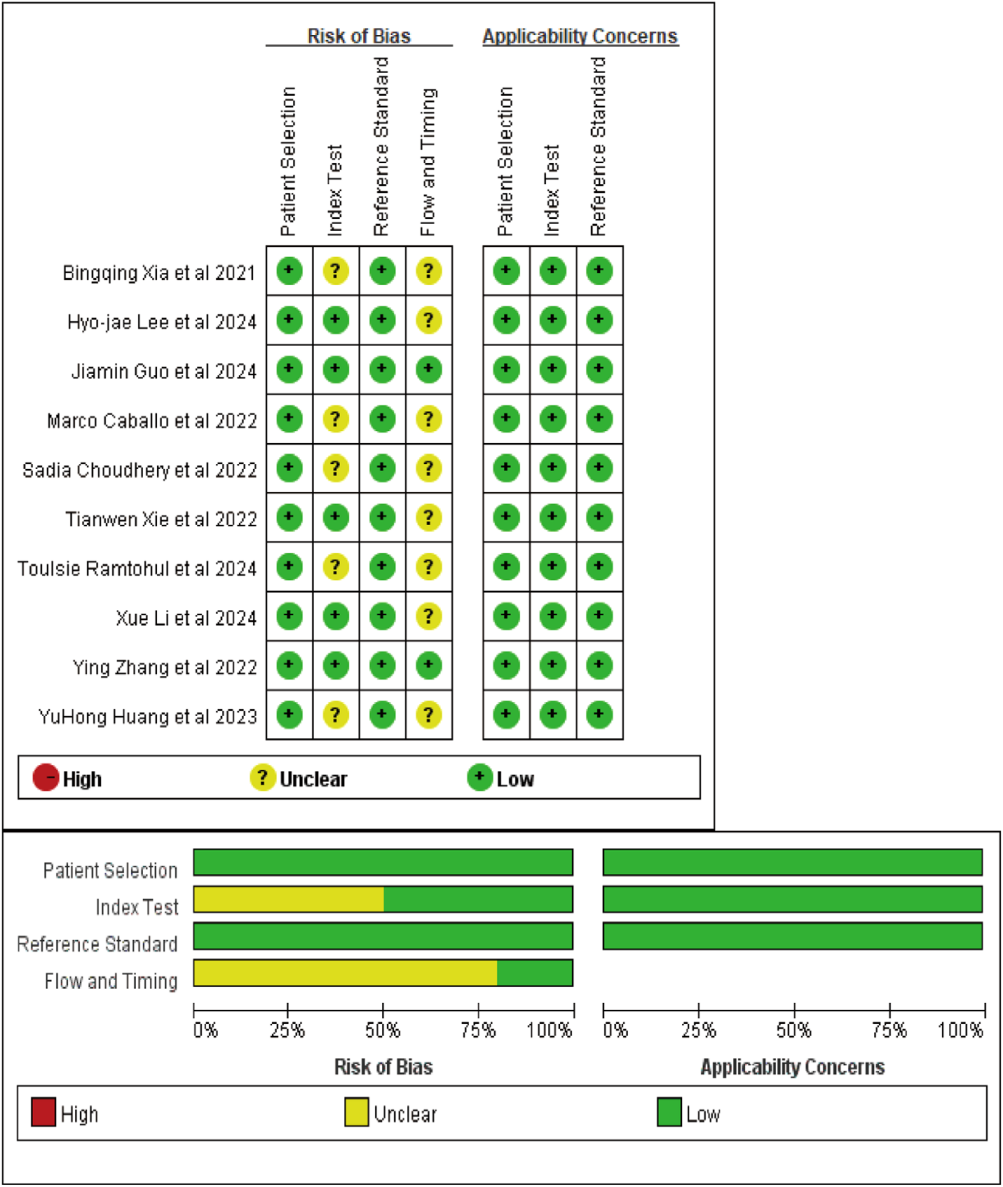
Methodologic quality of the included studies assessed according to the quality assessment of diagnostic accuracy study 2 tool for risk of bias and applicability concerns. green represents low, yellow unclear, and red high risk of bias.

QUADAS‐2 evaluation revealed overall good methodological quality across studies (Figure 2). All studies demonstrated low risk of bias in patient selection and reference standard application. While only four studies explicitly reported blinding,^35^, ^38^, ^39^, ^40^ implicit blinding was presumed as reference standards preceded model development. Threshold‐related bias was uniformly low. However, eight studies^33^, ^34^, ^36^, ^37^, ^38^, ^39^, ^41^, ^42^ showed unclear risk regarding time intervals between reference assessments.

### 3.4 Results of meta‐analysis

#### 3.4.1 Diagnostic value of MRI radiomics for predicting NAC efficacy

**FIGURE 3:**
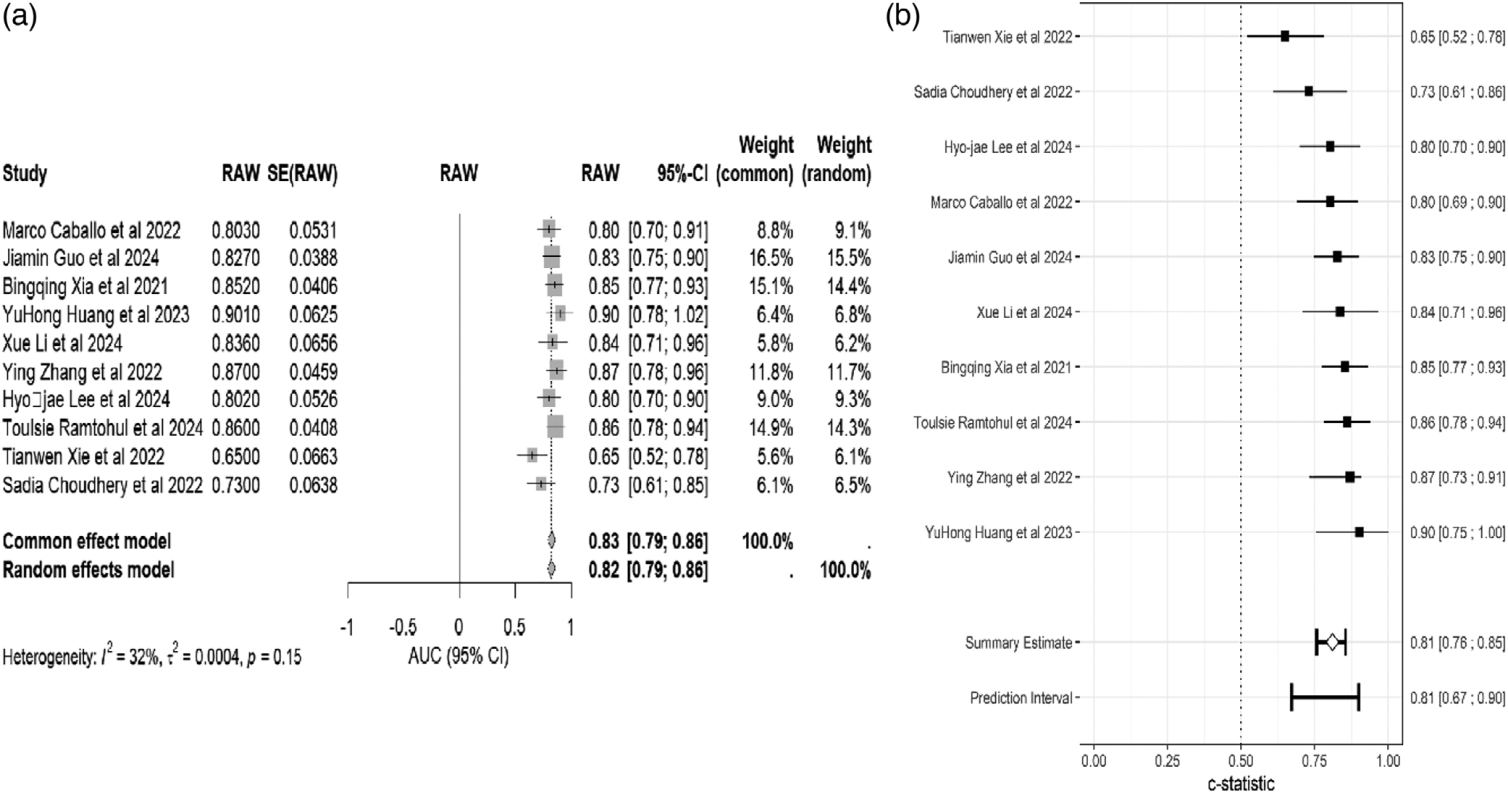
Forest plot: AUC of single studies and pooled area under the curve (AUC) and 95% CI of radiomic accuracy for predicting pathological complete response to neoadjuvant treatment. (A) Merging AUC forest plots using the metafor package. (B) Merging AUC forest plots using the metadisc package.

A secondary analysis of six studies^33^, ^36^, ^37^, ^38^, ^40^, ^42^ showed good performance: sensitivity of 0.80 (0.68–0.88), specificity of 0.85 (0.76–0.91), PLR of 5.36 (3.46–8.38), NLR of 0.24 (0.15–0.37), and DOR of 22.63 (14.40–0–35.58)(Table 3). A pooled analysis of 10 studies showed that the fixed‐effects model (*I* ^2^ = 32%, *p* = 0.15) demonstrated excellent predictive accuracy for NAC efficacy, with a pooled AUC of 0.83 (95% CI: 0.79–0.86)(Figure 3).

**TABLE 3:** Diagnostic value of mri radiomics for predicting NAC efficacy(*n* = 6).

Study (Author, year)	sensitivity (95% CI)	Specificity (95% CI)	positive likelihood ratios (95% CI)	negative likelihood ratios (95% CI)
Jiamin Guo et al. 2024	0.71 [0.5, 0.83]	0.85 [0.76, 0.91]	4.72 [2.74, 8.16]	0.35 [0.20, 0.59]
Bingqing Xia et al. 2021	0.56 [0.27, 0.81]	0.98 [0.87, 1]	22.78 [3.02, 172.08]	0.46 [0.22, 0.95]
YuHong Huang et al. 2023	0.86 [0.76, 0.93]	0.84 [0.78, 0.89]	5.43 [3.81, 7.73]	0.16 [0.09, 0.30]
Xue Li et al. 2024	0.70 [0.49, 0.84]	0.91 [0.81, 0.96]	7.54 [3.36, 16.92]	0.34 [0.18, 0.62]
Toulsie Ramtohul et al. 2024	0.88 [0.72, 0.95]	0.72 [0.63, 0.80]	3.14 [2.22, 4.44]	0.17 [0.07, 0.44]
Tianwen Xie et al. 2022	0.89 [0.78, 0.95]	0.69 [0.51, 0.83]	2.87 [1.66, 4.98]	0.16 [0.07, 0.35]

Abbreviation: CI, confidence interval.

#### 3.4.2 Meta‐regression analysis

We explored sources of heterogeneity despite *I* ^2^ (32%) being less than 50%. We analyzed covariate models for country, study type, field strength, validation (cross‐validation or test set), and pCR definition, and the *p*‐values of 0.996, 0.736, 0.597, and 0.917 were greater than 0.05, and there was no significant effect of heterogeneity for any of the five models (Table S2).

#### 3.4.3 Subgroup analysis

Our subgroup analysis of classifier types showed that machine learning methods significantly outperformed traditional methods, with SVM (AUC = 0.86, 95% CI 0.41–0.98) and LightGBM models (AUC = 0.86, 95% CI 0.52–0.97) showing higher diagnostic accuracy compared to LR (AUC = 0.79, 95% CI 0.68–0.89)) showed higher diagnostic accuracy. The results of the subgroup analysis are shown in Table 4.

**TABLE 4:** Subgroup analyses were performed on the combined AUC of the different classifiers.

Classifier	Study (Author, year)	AUC (95% CI)	Summary estimate (95% CI)
LR	Sadia Choudhery et al. (2022)	0.73 (0.61, 0.86)	0.79 (0.65, 0.89)
	Marco Caballo et al. (2022)	0.80 (0.69, 0.90)	
	Jiamin Guo et al. (2024)	0.83 (0.75, 0.90)	
SVM	Bingqing Xia et al. (2021)	0.85 (0.77, 0.93)	0.86 (0.41, 0.98)
	YuHong Huang et al. (2023)	0.90 (0.75, 1.00)	
LightGBM	Xue Li et al. (2024)	0.84 (0.71, 0.96)	0.86 (0.52, 0.97)
	Ying Zhang et al. (2022)	0.87 (0.73, 0.91)	
NR	Hyo‐jae Lee et al. (2024)	0.80 (0.70, 0.90)	0.83 (0.26, 0.99)
	Toulsie Ramtohul et al. (2024)	0.86 (0.78, 0.94)	
K‐NN	Tianwen Xie et al. (2022)	0.65 (0.52, 0.78)	0.65 (0.51, 0.77)
Overall			0.81 (0.76, 0.85)
			Prediction interval: 0.81 (0.67, 0.90)

Abbreviations: CI, confidence interval; K‐NN, K‐nearest neighbor; LightGBM, Light gradient boosting machine; LR, logistic regression; NR, not reported; SVM, super vector machine.

#### 3.4.4 Overall modeling and publication bias assessment

**FIGURE 4:**
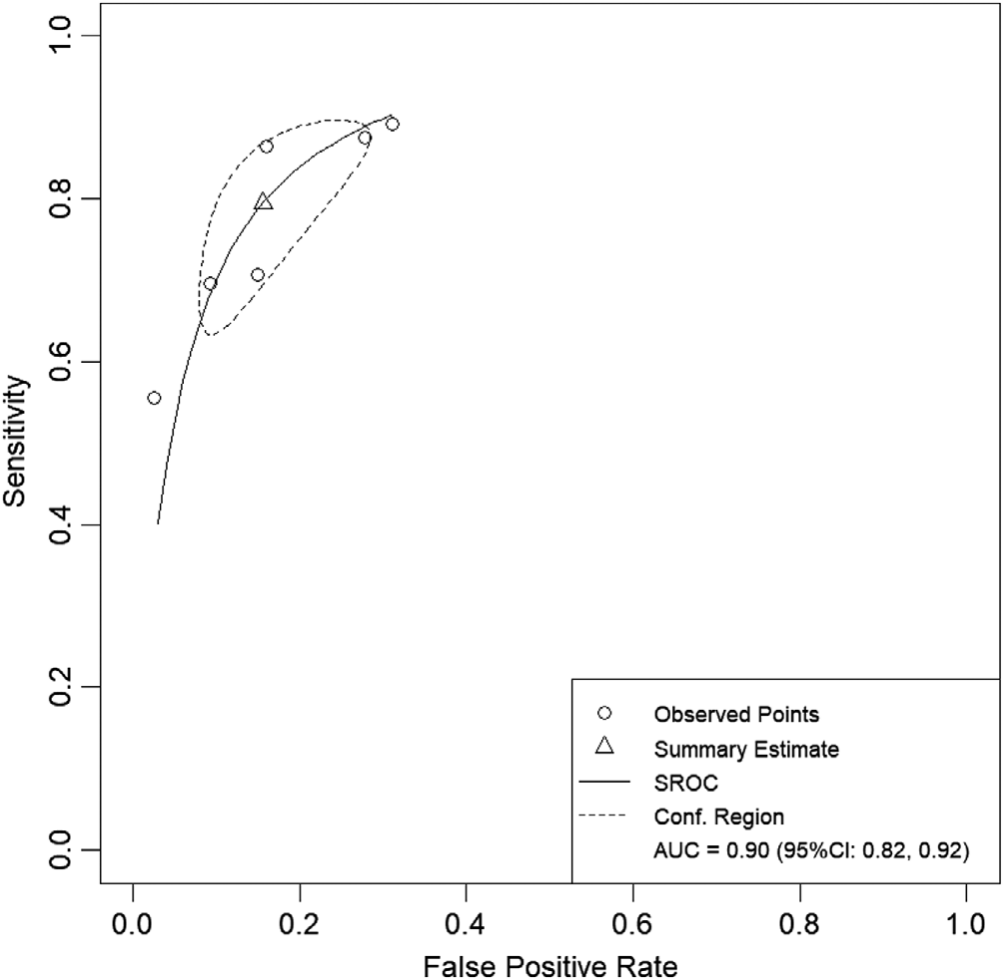
Summarized subject work characteristic curve (HSROC) for the entire machine learning model in the study. SROC, summary receiver operating characteristic; CI, confidence interval; AUC, area under the receiver operating characteristic curve.

**FIGURE 5:**
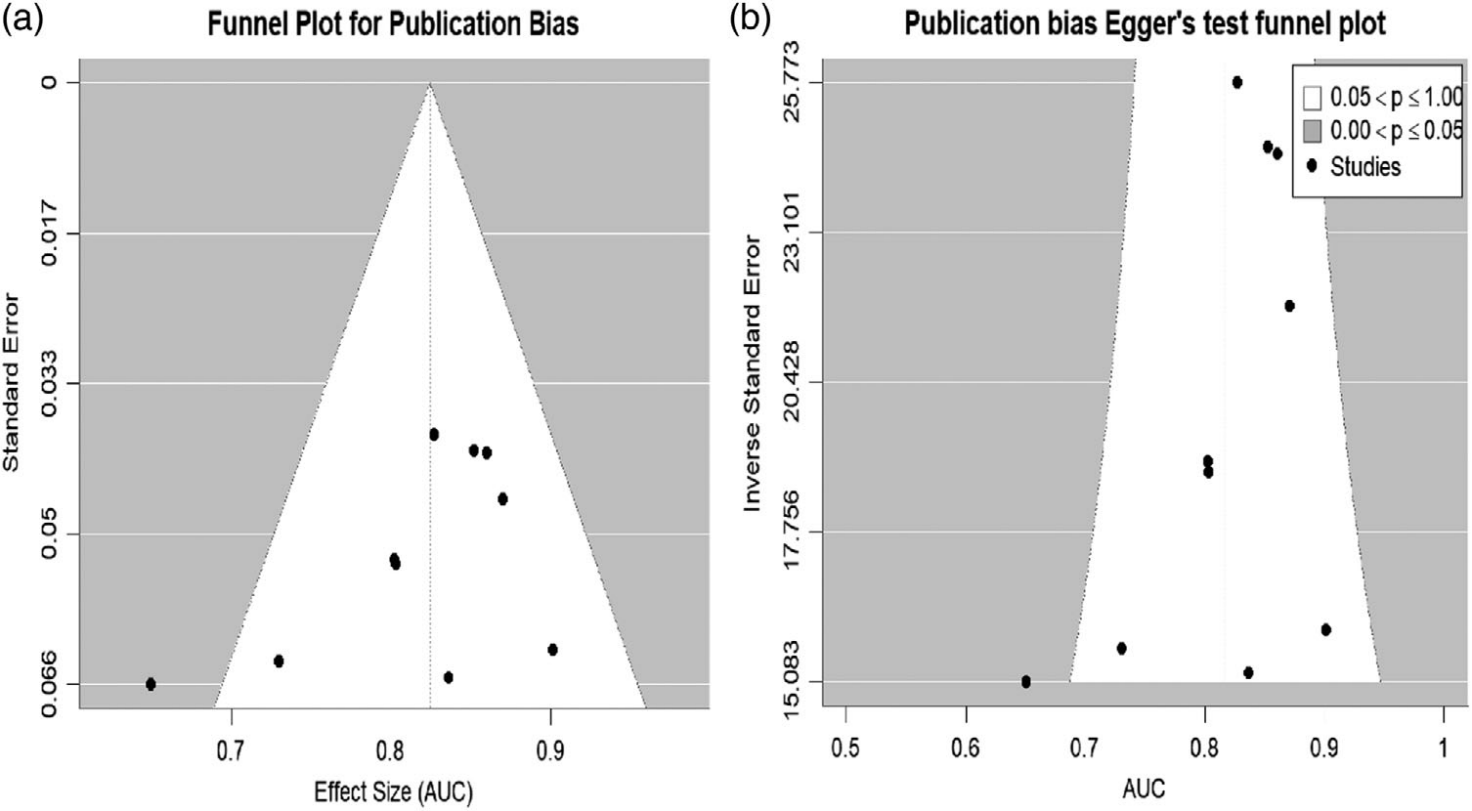
Publication bias analysis using Deek's funnel plot. (A) Deek^,^s funnel plot, (B) Egger's test.

The bivariate model analysis of six studies demonstrated excellent predictive performance, with a pooled AUC of 0.90 (95% CI: 0.82–0.92) for the imaging‐histologic model Figure 4. Publication bias assessment revealed no significant bias, as evidenced by a symmetrical funnel plot (Deek's test) and non‐significant Egger's test result (*p* = 0.064, Figure 5).

## 4 DISCUSSION

### 4.1 Principal findings

In this study, MRI‐based radiomics predicting pCR in TNBC patients treated with NAC was systematically assessed by two independent reviewers using the Radiomics Quality Score (RQS), which obtained a very high inter‐rater reliability (Cohen's κ = 0.96, 95% CI 0.86–0.99). Publication bias was comprehensively assessed by funnel plot asymmetry test and Egger regression (*p* = 0.064), which showed no significant evidence of selective reporting bias among the 10 included studies.

The meta‐analysis showed that the predictive performance of the different models was strong: AUC = 0.83 (95% CI 0.79–0.86) for the fixed‐effects model, AUC = 0.82 (95% CI 0.79–0.86) for the random‐effects model, and AUC = 0.81 (95% CI 0.76–0.85) for the pooled AUC. Sensitivity/specificity analyses showed significant diagnostic utility with pooled estimates of 0.80 (0.68–0.88) and 0.85 (0.76–0.91), respectively (PLR = 5.36, NLR = 0.24, DOR = 22.63). Subgroup analysis identified SVM combined with LightGBM as the best classifier (AUC = 0.86), while meta‐regression confirmed that there were no significant heterogeneous effects among the five covariates (*p* > 0.05).

### 4.2 Comparison with other meta‐analysis

Our findings demonstrate that MRI‐based radiomics provides superior predictive performance for pCR in TNBC patients compared to conventional imaging assessment methods. While previous meta‐analyses have primarily focused on pCR rates^43^ and chemotherapy regimen efficacy,^44^ our study represents the first comprehensive evaluation of radiomics‐based prediction models in this clinical context. The pooled AUC of 0.83 (95% CI: 0.79–0.86) in our analysis compares favorably with the diagnostic accuracy reported in ultrasound‐based radiomics studies (AUC: 0.75–0.82),^44^ suggesting that MRI radiomics may offer enhanced predictive capability for treatment response assessment.

Subgroup analyses revealed important insights into model performance characteristics. Notably, the SVM and LightGBM classifiers had the highest diagnostic validity (AUC = 0.86), outperforming the logistic regression model (AUC = 0.79). This finding is consistent with performance differences in previous studies,^20^, ^21^ but methodological differences need to be interpreted with caution.

Our results also provide context for understanding the relationship between chemotherapy regimens and predictive accuracy. Studies incorporating platinum‐based regimens demonstrated higher AUC values, consistent with previous reports of improved pCR rates with platinum‐containing regimens (OR = 2.16, 95% CI:1.20–3.91).^44^ This observation suggests that radiomics features may capture distinct tumor biological characteristics that influence both chemotherapy sensitivity and imaging phenotype.

### 4.3 limitations

This study has several limitations. First, despite an extensive search using both free‐text and subject terms to identify relevant studies, only 10 articles met the inclusion criteria after screening. Because only six studies reported all of the basic diagnostic accuracy metrics required for robust sensitivity/specificity analyses, secondary analyses were performed. In contrast, the other four studies underwent pooled AUC analysis only. As the field of radiomics advances, we plan to update this review with additional studies in the future. Second, the included studies exhibited some heterogeneity in their AUC values. Although heterogeneity was low, meta‐regression analyses were conducted to identify potential sources, but no significant factors were found (*p* > 0.05). Other factors, such as variations in imaging devices and feature extraction methods, may contribute to the observed heterogeneity. Third, we have analyzed the data statistically using *R*. However, the results varied slightly depending on the *R* package used. This may be due to the different algorithms utilized in them. Fourth, the studies were conducted in single‐center hospital settings with limited training and testing data, which may reduce statistical validity and generalizability. Fifth, our analysis focuses on the highest reported AUC value to assess the upper limit of diagnostic performance, which may introduce some optimism bias. Finally, the included studies were conducted in various countries, including Sweden, China, USA, South Korea, and France. However, there are various differences between different countries and regions. Differences in demographic factors, such as age distribution, health status, and genetic background, may influence disease onset, progression, and diagnostic accuracy. Variations in medical resources and their distribution may result in disparities in healthcare quality, impacting treatment evaluation. Therefore, meta‐analysis results should be interpreted with caution, considering regional differences and potential limitations in generalizability.

### 4.4 clinical significants and perspectives for research

This study demonstrates that MRI‐based radiomics has high accuracy in predicting pathological complete response to neoadjuvant chemotherapy in TNBC, and that machine learning classifiers (SVM/LightGBM) show better promise. Despite the promising results of these studies, some limitations need to be noted in future studies. The retrospective design of most of the included studies introduces inherent biases related to patient selection and treatment regimens, especially given the heterogeneity of NAC regimens and pCR definitions.^37^ In addition, the lack of prospective validation^37^ and multicenter collaborations limit the generalizability of the results across different populations and imaging platforms. Bridging these gaps requires standardized workflows such as adherence to the Image Biomarker Standardization Initiative (IBSI)^22^ and integration of multimodal data.^23^ Notably, cost‐effectiveness analyses are still conspicuously absent, which is a key obstacle to clinical application given the resource‐intensive nature of the radiomics pipeline.

## 5 CONCLUSIONS

MRI‐based radiomics is a valuable tool for evaluating TNBC response to NAC.^37^, ^41^ However, the application of radiomics as a clinical biomarker still needs to be improved in terms of prospective assessment, reproducibility and standardization before it can be applied to routine clinical practice.

## AUTHOR CONTRIBUTION

XZ and BL contributed to the study concept and design. JZ and QW retrieved and filtered the articles. JP and PL extracted the data. JZ and QW analyzed the data. JZ, QW, and PL interpreted the data. JZ and QW drafted the manuscript. XZ and BL contributed to the critical revision of the manuscript. All authors contributed to the paper and approved the submitted version.

## CONFLICT OF INTEREST STATEMENT

The authors declare no conflicts of interest.

## Supporting information



Supporting Information

## Data Availability

The raw data supporting the conclusions of this article will be made available by the authors, without undue reservation. All relevant data are within the article and its Supporting Information files.
