# Supplementary material for: MRI‐based radiomics models for early predicting pathological response to neoadjuvant chemotherapy in triple‐negative breast cancer: A systematic review and meta‐analysis
Source: J Appl Clin Med Phys. 2025 Oct 15;26(10):e70296. doi: 10.1002/acm2.70296 (PMC12527642; doi:10.1002/acm2.70296)
Supplement: Supplementary file 1 — Supporting Information [file ACM2-26-e70296-s001.docx]

**Search Strategy**

**Publication of search results is limited to the period from inception until September 2024**

**1.The search formula for retrieving PubMed is as follows:**

("Triple Negative Breast Neoplasms"[MeSH Terms] OR ("Triple Negative Breast Neoplasms"[MeSH Terms] OR ("triple"[All Fields] AND "negative"[All Fields] AND "breast"[All Fields] AND "neoplasms"[All Fields]) OR "Triple Negative Breast Neoplasms"[All Fields] OR ("er"[All Fields] AND "negative"[All Fields] AND "pr"[All Fields] AND "her2"[All Fields] AND "breast"[All Fields] AND "cancer"[All Fields]) OR "er negative pr negative her2 negative breast cancer"[All Fields] OR ("Triple Negative Breast Neoplasms"[MeSH Terms] OR ("triple"[All Fields] AND "negative"[All Fields] AND "breast"[All Fields] AND "neoplasms"[All Fields]) OR "Triple Negative Breast Neoplasms"[All Fields] OR ("er"[All Fields] AND "negative"[All Fields] AND "pr"[All Fields] AND "her2"[All Fields] AND "breast"[All Fields] AND "cancer"[All Fields]) OR "er negative pr negative her2 negative breast cancer"[All Fields]) OR ("Triple Negative Breast Neoplasms"[MeSH Terms] OR ("triple"[All Fields] AND "negative"[All Fields] AND "breast"[All Fields] AND "neoplasms"[All Fields]) OR "Triple Negative Breast Neoplasms"[All Fields] OR ("er"[All Fields] AND "negative"[All Fields] AND "pr"[All Fields] AND "her2"[All Fields] AND "breast"[All Fields] AND "neoplasms"[All Fields])) OR ("Triple Negative Breast Neoplasms"[MeSH Terms] OR ("triple"[All Fields] AND "negative"[All Fields] AND "breast"[All Fields] AND "neoplasms"[All Fields]) OR "Triple Negative Breast Neoplasms"[All Fields] OR ("er"[All Fields] AND "negative"[All Fields] AND "pr"[All Fields] AND "her2"[All Fields] AND "breast"[All Fields] AND "neoplasms"[All Fields])) OR ("Triple Negative Breast Neoplasms"[MeSH Terms] OR ("triple"[All Fields] AND "negative"[All Fields] AND "breast"[All Fields] AND "neoplasms"[All Fields]) OR "Triple Negative Breast Neoplasms"[All Fields] OR ("triple"[All Fields] AND "negative"[All Fields] AND "breast"[All Fields] AND "cancer"[All Fields]) OR "triple negative breast cancer"[All Fields]) OR ("Triple Negative Breast Neoplasms"[MeSH Terms] OR ("triple"[All Fields] AND "negative"[All Fields] AND "breast"[All Fields] AND "neoplasms"[All Fields]) OR "Triple Negative Breast Neoplasms"[All Fields] OR ("triple"[All Fields] AND "negative"[All Fields] AND "breast"[All Fields] AND "cancer"[All Fields]) OR "triple negative breast cancer"[All Fields]) OR (("breast neoplasms"[MeSH Terms] OR ("breast"[All Fields] AND "neoplasms"[All Fields]) OR "breast neoplasms"[All Fields] OR ("breast"[All Fields] AND "cancers"[All Fields]) OR "breast cancers"[All Fields]) AND "Triple-Negative"[All Fields]) OR (("breast neoplasms"[MeSH Terms] OR ("breast"[All Fields] AND "neoplasms"[All Fields]) OR "breast neoplasms"[All Fields] OR ("breast"[All Fields] AND "cancer"[All Fields]) OR "breast cancer"[All Fields]) AND "Triple-Negative"[All Fields]) OR ("Triple Negative Breast Neoplasms"[MeSH Terms] OR ("triple"[All Fields] AND "negative"[All Fields] AND "breast"[All Fields] AND "neoplasms"[All Fields]) OR "Triple Negative Breast Neoplasms"[All Fields] OR ("triple"[All Fields] AND "negative"[All Fields] AND "breast"[All Fields] AND "cancers"[All Fields]) OR "triple negative breast cancers"[All Fields]) OR ("Triple Negative Breast Neoplasms"[MeSH Terms] OR ("triple"[All Fields] AND "negative"[All Fields] AND "breast"[All Fields] AND "neoplasms"[All Fields]) OR "Triple Negative Breast Neoplasms"[All Fields] OR ("triple"[All Fields] AND "negative"[All Fields] AND "breast"[All Fields] AND "neoplasm"[All Fields]) OR "triple negative breast neoplasm"[All Fields]) OR (("breast neoplasms"[MeSH Terms] OR ("breast"[All Fields] AND "neoplasms"[All Fields]) OR "breast neoplasms"[All Fields]) AND "Triple-Negative"[All Fields]) OR (("breast neoplasms"[MeSH Terms] OR ("breast"[All Fields] AND "neoplasms"[All Fields]) OR "breast neoplasms"[All Fields] OR ("breast"[All Fields] AND "neoplasm"[All Fields]) OR "breast neoplasm"[All Fields]) AND "Triple-Negative"[All Fields]) OR ("Triple Negative Breast Neoplasms"[MeSH Terms] OR ("triple"[All Fields] AND "negative"[All Fields] AND "breast"[All Fields] AND "neoplasms"[All Fields]) OR "Triple Negative Breast Neoplasms"[All Fields] OR ("triple"[All Fields] AND "negative"[All Fields] AND "breast"[All Fields] AND "neoplasm"[All Fields]) OR "triple negative breast neoplasm"[All Fields]) OR ("Triple Negative Breast Neoplasms"[MeSH Terms] OR ("triple"[All Fields] AND "negative"[All Fields] AND "breast"[All Fields] AND "neoplasms"[All Fields]) OR "Triple Negative Breast Neoplasms"[All Fields]))) AND ("Radiomics"[MeSH Terms] OR "Radiomics"[All Fields] OR "radiomic"[All Fields] OR "Radiomics"[MeSH Terms])

**2. The search formula for searching Scopus is as follows:**

( ( TITLE-ABS-KEY ( radiomics ) OR TITLE-ABS-KEY ( radiomic ) ) ) AND ( ( TITLE-ABS-KEY ( neoadjuvant AND therapy ) OR TITLE-ABS-KEY ( neoadjuvant AND therapies ) OR TITLE-ABS-KEY ( therapy, AND neoadjuvant ) OR TITLE-ABS-KEY ( neoadjuvant AND treatment ) OR TITLE-ABS-KEY ( neoadjuvant AND treatments ) OR TITLE-ABS-KEY ( treatment, AND neoadjuvant ) OR TITLE-ABS-KEY ( neoadjuvant AND chemotherapy ) OR TITLE-ABS-KEY ( chemotherapy, AND neoadjuvant ) OR TITLE-ABS-KEY ( neoadjuvant AND chemotherapies ) OR TITLE-ABS-KEY ( neoadjuvant AND chemotherapy AND treatment ) OR TITLE-ABS-KEY ( chemotherapy AND treatment, AND neoadjuvant ) OR TITLE-ABS-KEY ( neoadjuvant AND chemotherapy AND treatments ) OR TITLE-ABS-KEY ( treatment, AND neoadjuvant AND chemotherapy ) OR TITLE-ABS-KEY ( neoadjuvant AND chemoradiotherapy ) OR TITLE-ABS-KEY ( chemoradiotherapy, AND neoadjuvant ) OR TITLE-ABS-KEY ( neoadjuvant AND chemoradiotherapies ) OR TITLE-ABS-KEY ( neoadjuvant AND chemoradiation ) OR TITLE-ABS-KEY ( chemoradiation, AND neoadjuvant ) OR TITLE-ABS-KEY ( neoadjuvant AND chemoradiations ) OR TITLE-ABS-KEY ( neoadjuvant AND chemoradiation AND therapy ) OR TITLE-ABS-KEY ( chemoradiation AND therapy, AND neoadjuvant ) OR TITLE-ABS-KEY ( neoadjuvant AND chemoradiation AND therapies ) OR TITLE-ABS-KEY ( therapy, AND neoadjuvant AND chemoradiation ) OR TITLE-ABS-KEY ( neoadjuvant AND chemoradiation AND treatment ) OR TITLE-ABS-KEY ( chemoradiation AND treatment, AND neoadjuvant ) OR TITLE-ABS-KEY ( treatment, AND neoadjuvant AND chemoradiation ) OR TITLE-ABS-KEY ( neoadjuvant AND systemic AND therapy ) OR TITLE-ABS-KEY ( neoadjuvant AND systemic AND therapies ) OR TITLE-ABS-KEY ( systemic AND therapy, AND neoadjuvant ) OR TITLE-ABS-KEY ( therapy, AND neoadjuvant AND systemic ) OR TITLE-ABS-KEY ( neoadjuvant AND systemic AND treatment ) OR TITLE-ABS-KEY ( neoadjuvant AND systemic AND treatments ) OR TITLE-ABS-KEY ( systemic AND treatment, AND neoadjuvant ) OR TITLE-ABS-KEY ( treatment, AND neoadjuvant AND systemic ) OR TITLE-ABS-KEY ( neoadjuvant AND radiotherapy ) OR TITLE-ABS-KEY ( neoadjuvant AND radiotherapies ) OR TITLE-ABS-KEY ( radiotherapy, AND neoadjuvant ) OR TITLE-ABS-KEY ( neoadjuvant AND radiation ) OR TITLE-ABS-KEY ( neoadjuvant AND radiations ) OR TITLE-ABS-KEY ( radiation, AND neoadjuvant ) OR TITLE-ABS-KEY ( neoadjuvant AND radiation AND treatment ) OR TITLE-ABS-KEY ( neoadjuvant AND radiation AND treatments ) OR TITLE-ABS-KEY ( radiation AND treatment, AND neoadjuvant ) OR TITLE-ABS-KEY ( treatment, AND neoadjuvant AND radiation ) OR TITLE-ABS-KEY ( neoadjuvant AND radiation AND therapy ) OR TITLE-ABS-KEY ( neoadjuvant AND radiation AND therapies ) OR TITLE-ABS-KEY ( radiation AND therapy, AND neoadjuvant ) OR TITLE-ABS-KEY ( therapy, AND neoadjuvant AND radiation ) ) ) AND ( ( TITLE-ABS-KEY ( triple AND negative AND breast AND neoplasms ) OR TITLE-ABS-KEY ( er-negative AND pr-negative AND her2-negative AND breast AND cancer ) OR TITLE-ABS-KEY ( er AND negative AND pr AND negative AND her2 AND negative AND breast AND cancer ) OR TITLE-ABS-KEY ( er-negative AND pr-negative AND her2-negative AND breast AND neoplasms ) OR TITLE-ABS-KEY ( er AND negative AND pr AND negative AND her2 AND negative AND breast AND neoplasms ) OR TITLE-ABS-KEY ( triple AND negative AND breast AND cancer ) OR TITLE-ABS-KEY ( triple-negative AND breast AND cancer ) OR TITLE-ABS-KEY ( breast AND cancers, AND triple-negative ) OR TITLE-ABS-KEY ( breast AND cancer, AND triple-negative ) OR TITLE-ABS-KEY ( triple-negative AND breast AND cancers ) OR TITLE-ABS-KEY ( triple-negative AND breast AND neoplasm ) OR TITLE-ABS-KEY ( breast AND neoplasms, AND triple-negative ) OR TITLE-ABS-KEY ( breast AND neoplasm, AND triple-negative ) OR TITLE-ABS-KEY ( triple AND negative AND breast AND neoplasm ) OR TITLE-ABS-KEY ( triple-negative AND breast AND neoplasms ) ) )

**3. The search formula for retrieving Embase is as follows:**

'neoadjuvant therapy'/exp OR 'neoadjuvant therapy' OR 'neoadjuvant therapies' OR 'therapy, neoadjuvant' OR 'neoadjuvant treatment'/exp OR 'neoadjuvant treatment' OR 'neoadjuvant treatments' OR 'treatment, neoadjuvant' OR 'neoadjuvant chemotherapy'/exp OR 'neoadjuvant chemotherapy' OR 'chemotherapy, neoadjuvant' OR 'neoadjuvant chemotherapies' OR 'neoadjuvant chemotherapy treatment' OR 'chemotherapy treatment, neoadjuvant' OR 'neoadjuvant chemotherapy treatments' OR 'treatment, neoadjuvant chemotherapy' OR 'neoadjuvant chemoradiotherapy'/exp OR 'neoadjuvant chemoradiotherapy' OR 'chemoradiotherapy, neoadjuvant' OR 'neoadjuvant chemoradiotherapies' OR 'neoadjuvant chemoradiation' OR 'chemoradiation, neoadjuvant' OR 'neoadjuvant chemoradiations' OR 'neoadjuvant chemoradiation therapy'/exp OR 'neoadjuvant chemoradiation therapy' OR 'chemoradiation therapy, neoadjuvant' OR 'neoadjuvant chemoradiation therapies' OR 'therapy, neoadjuvant chemoradiation' OR 'neoadjuvant chemoradiation treatment' OR 'chemoradiation treatment, neoadjuvant' OR 'treatment, neoadjuvant chemoradiation' OR 'neoadjuvant systemic therapy'/exp OR 'neoadjuvant systemic therapy' OR 'neoadjuvant systemic therapies' OR 'systemic therapy, neoadjuvant' OR 'therapy, neoadjuvant systemic' OR 'neoadjuvant systemic treatment' OR 'neoadjuvant systemic treatments' OR 'systemic treatment, neoadjuvant' OR 'treatment, neoadjuvant systemic' OR 'neoadjuvant radiotherapy'/exp OR 'neoadjuvant radiotherapy' OR 'neoadjuvant radiotherapies' OR 'radiotherapy, neoadjuvant' OR 'neoadjuvant radiation' OR 'neoadjuvant radiations' OR 'radiation, neoadjuvant' OR 'neoadjuvant radiation treatment' OR 'neoadjuvant radiation treatments' OR 'radiation treatment, neoadjuvant' OR 'treatment, neoadjuvant radiation' OR 'neoadjuvant radiation therapy' OR 'neoadjuvant radiation therapies' OR 'radiation therapy, neoadjuvant' OR 'therapy, neoadjuvant radiation' AND 'radiomics'/exp OR 'radiomics' OR 'radiomic' AND 'triple negative breast neoplasms'/exp OR 'triple negative breast neoplasms' OR 'er-negative pr-negative her2-negative breast cancer' OR 'er negative pr negative her2 negative breast cancer' OR 'er-negative pr-negative her2-negative breast neoplasms' OR 'er negative pr negative her2 negative breast neoplasms' OR 'triple negative breast cancer'/exp OR 'triple negative breast cancer' OR 'triple-negative breast cancer'/exp OR 'triple-negative breast cancer' OR 'breast cancers, triple-negative' OR 'breast cancer, triple-negative' OR 'triple-negative breast cancers'/exp OR 'triple-negative breast cancers' OR 'triple-negative breast neoplasm'/exp OR 'triple-negative breast neoplasm' OR 'breast neoplasms, triple-negative' OR 'breast neoplasm, triple-negative' OR 'triple negative breast neoplasm'/exp OR 'triple negative breast neoplasm' OR 'triple-negative breast neoplasms'/exp OR 'triple-negative breast neoplasms'

**4.The search formula for searching Cochrane is as follows:**

(‘Neoadjuvant Therapy’ OR ‘Neoadjuvant Therapies’ OR ‘Therapy, Neoadjuvant’ OR ‘Neoadjuvant Treatment’ OR ‘Neoadjuvant Treatments’ OR ‘Treatment, Neoadjuvant’ OR ‘Neoadjuvant Chemotherapy’ OR ‘Chemotherapy, Neoadjuvant’ OR ‘Neoadjuvant Chemotherapies’ OR ‘Neoadjuvant Chemotherapy Treatment’ OR ‘Chemotherapy Treatment, Neoadjuvant’ OR ‘Neoadjuvant Chemotherapy Treatments’ OR ‘Treatment, Neoadjuvant Chemotherapy’ OR ‘Neoadjuvant Chemoradiotherapy’ OR ‘Chemoradiotherapy, Neoadjuvant’ OR ‘Neoadjuvant Chemoradiotherapies’ OR ‘Neoadjuvant Chemoradiation’ OR ‘Chemoradiation, Neoadjuvant’ OR ‘Neoadjuvant Chemoradiations’ OR ‘Neoadjuvant Chemoradiation Therapy’ OR ‘Chemoradiation Therapy, Neoadjuvant’ OR ‘Neoadjuvant Chemoradiation Therapies’ OR ‘Therapy, Neoadjuvant Chemoradiation’ OR ‘Neoadjuvant Chemoradiation Treatment’ OR ‘Chemoradiation Treatment, Neoadjuvant’ OR ‘Treatment, Neoadjuvant Chemoradiation’ OR ‘Neoadjuvant Systemic Therapy’ OR ‘Neoadjuvant Systemic Therapies’ OR ‘Systemic Therapy, Neoadjuvant’ OR ‘Therapy, Neoadjuvant Systemic’ OR ‘Neoadjuvant Systemic Treatment’ OR ‘Neoadjuvant Systemic Treatments’ OR ‘Systemic Treatment, Neoadjuvant’ OR ‘Treatment, Neoadjuvant Systemic’ OR ‘Neoadjuvant Radiotherapy’ OR ‘Neoadjuvant Radiotherapies’ OR ‘Radiotherapy, Neoadjuvant’ OR ‘Neoadjuvant Radiation’ OR ‘Neoadjuvant Radiations’ OR ‘Radiation, Neoadjuvant’ OR ‘Neoadjuvant Radiation Treatment’ OR ‘Neoadjuvant Radiation Treatments’ OR ‘Radiation Treatment, Neoadjuvant’ OR ‘Treatment, Neoadjuvant Radiation’ OR ‘Neoadjuvant Radiation Therapy’ OR ‘Neoadjuvant Radiation Therapies’ OR ‘Radiation Therapy, Neoadjuvant’ OR ‘Therapy, Neoadjuvant Radiation’):ti,ab,kw

(’Radiomics‘ OR ‘Radiomic’):ti,ab,kw

(Triple Negative Breast Cancer OR Triple-Negative Breast Cancer OR Breast Cancers, Triple-Negative OR Breast Cancer, Triple-Negative OR Triple-Negative Breast Cancers OR Triple-Negative Breast Neoplasm OR Breast Neoplasms, Triple-Negative OR Breast Neoplasm, Triple-Negative OR Triple Negative Breast Neoplasm OR Triple-Negative Breast Neoplasms):ti,ab,kw

**5. The search formula for retrieving web of science is as follows:**

(TS=(Radiomics) OR TS=(Radiomic)) NOT (SILOID==("PPRN")) AND (TS=(Neoadjuvant Therapy) OR TS=(Neoadjuvant Therapies) OR TS=(Therapy, Neoadjuvant) OR TS=(Neoadjuvant Treatment) OR TS=(Neoadjuvant Treatments) OR TS=(Treatment, Neoadjuvant) OR TS=(Neoadjuvant Chemotherapy) OR TS=(Chemotherapy, Neoadjuvant) OR TS=(Neoadjuvant Chemotherapies) OR TS=(Neoadjuvant Chemotherapy Treatment) OR TS=(Chemotherapy Treatment, Neoadjuvant) OR TS=(Neoadjuvant Chemotherapy Treatments) OR TS=(Treatment, Neoadjuvant Chemotherapy) OR TS=(Neoadjuvant Chemoradiotherapy) OR TS=(Chemoradiotherapy, Neoadjuvant) OR TS=(Neoadjuvant Chemoradiotherapies) OR TS=(Neoadjuvant Chemoradiation) OR TS=(Chemoradiation, Neoadjuvant) OR TS=(Neoadjuvant Chemoradiations) OR TS=(Neoadjuvant Chemoradiation Therapy) OR TS=(Chemoradiation Therapy, Neoadjuvant) OR TS=(Neoadjuvant Chemoradiation Therapies) OR TS=(Therapy, Neoadjuvant Chemoradiation) OR TS=(Neoadjuvant Chemoradiation Treatment) OR TS=(Chemoradiation Treatment, Neoadjuvant) OR TS=(Treatment, Neoadjuvant Chemoradiation) OR TS=(Neoadjuvant Systemic Therapy) OR TS=(Neoadjuvant Systemic Therapies) OR TS=(Systemic Therapy, Neoadjuvant) OR TS=(Therapy, Neoadjuvant Systemic) OR TS=(Neoadjuvant Systemic Treatment) OR TS=(Neoadjuvant Systemic Treatments) OR TS=(Systemic Treatment, Neoadjuvant) OR TS=(Treatment, Neoadjuvant Systemic) OR TS=(Neoadjuvant Radiotherapy) OR TS=(Neoadjuvant Radiotherapies) OR TS=(Radiotherapy, Neoadjuvant) OR TS=(Neoadjuvant Radiation) OR TS=(Neoadjuvant Radiations) OR TS=(Radiation, Neoadjuvant) OR TS=(Neoadjuvant Radiation Treatment) OR TS=(Neoadjuvant Radiation Treatments) OR TS=(Radiation Treatment, Neoadjuvant) OR TS=(Treatment, Neoadjuvant Radiation) OR TS=(Neoadjuvant Radiation Therapy) OR TS=(Neoadjuvant Radiation Therapies) OR TS=(Radiation Therapy, Neoadjuvant) OR TS=(Therapy, Neoadjuvant Radiation)) NOT (SILOID==("PPRN")) AND (TS=(Triple Negative Breast Neoplasms ) OR TS=(ER-Negative PR-Negative HER2-Negative Breast Cancer) OR TS=(ER Negative PR Negative HER2 Negative Breast Cancer ) OR TS=(ER-Negative PR-Negative HER2-Negative Breast Neoplasms) OR TS=(ER Negative PR Negative HER2 Negative Breast Neoplasms ) OR TS=(Triple Negative Breast Cancer) OR TS=(Triple-Negative Breast Cancer) OR TS=(Breast Cancers, Triple-Negative ) OR TS=(Breast Cancer, Triple-Negative) OR TS=(Triple-Negative Breast Cancers ) OR TS=(Triple-Negative Breast Neoplasm) OR TS=(Breast Neoplasms, Triple-Negative) OR TS=(Breast Neoplasm, Triple-Negative ) OR TS=(Triple Negative Breast Neoplasm) OR TS=(Triple-Negative Breast Neoplasms )) NOT (SILOID==("PPRN"))

**Table S1 Below are the radiomics scores for each study.**

| **Authors** | **Year** | **Tot** | **RQS_1** | **RQS_2** | **RQS_3** | **RQS_4** | **RQS_5** | **RQS_6** | **RQS_7** | **RQS_8** | **RQS_9** | **RQS_10** | **RQS_11** | **RQS_12** | **RQS_13** | **RQS_14** | **RQS_15** | **RQS_16** |
| --- | --- | --- | --- | --- | --- | --- | --- | --- | --- | --- | --- | --- | --- | --- | --- | --- | --- | --- |
| Marco Caballo et al | 2022 | 13 | 1 | 1 | 0 | 0 | 3 | 0 | 1 | 0 | 1 | 0 | 0 | 2 | 2 | 0 | 0 | 2 |
| Jiamin Guo et al | 2024 | 16 | 1 | 1 | 0 | 0 | 3 | 0 | 0 | 0 | 1 | 1 | 0 | 3 | 2 | 2 | 0 | 2 |
| Bingqing Xia et al | 2021 | 14 | 1 | 1 | 0 | 0 | 3 | 0 | 0 | 0 | 1 | 0 | 0 | 4 | 2 | 0 | 0 | 2 |
| YuHong Huang et al | 2023 | 21 | 1 | 1 | 0 | 0 | 3 | 0 | 1 | 0 | 1 | 1 | 0 | 5 | 2 | 2 | 0 | 4 |
| Xue Li et al | 2024 | 12 | 1 | 1 | 0 | 0 | 3 | 0 | 0 | 0 | 1 | 0 | 0 | 2 | 2 | 0 | 0 | 2 |
| Choudhery et al | 2022 | 11 | 1 | 1 | 0 | 0 | 3 | 0 | 0 | 0 | 1 | 0 | 0 | 2 | 2 | 0 | 0 | 1 |
| Ying Zhang et | 2022 | 15 | 1 | 1 | 0 | 0 | 3 | 1 | 1 | 0 | 1 | 0 | 0 | 2 | 2 | 0 | 0 | 2 |
| Hyo‑jae Lee et al | 2024 | 13 | 1 | 1 | 0 | 0 | 3 | 0 | 1 | 0 | 1 | 0 | 0 | 2 | 2 | 0 | 0 | 2 |
| Toulsie Ramtohul et al | 2024 | 25 | 1 | 1 | 0 | 0 | 3 | 0 | 1 | 0 | 1 | 1 | 7 | 3 | 2 | 2 | 0 | 3 |
| Tianwen Xie et al | 2022 | 15 | 1 | 1 | 0 | 0 | 3 | 0 | 1 | 0 | 1 | 0 | 0 | 4 | 2 | 0 | 0 | 2 |

| Variable Name | Coefficient | Standard Error | t-Value | P-Value | 95% Confidence Interval |
| --- | --- | --- | --- | --- | --- |
| country | 0.0003076 | 0.0626607 | 0.00 | 0.996 | (-0.1991067, 0.1997218) |
| Type of study | 0.0446143 | 0.1205599 | 0.37 | 0.736 | (-0.3390612, 0.4282899) |
| magnetic field strength | -0.0116903 | 0.0771012 | -0.15 | 0.889 | (-0.2570608, 0.2336802) |
| Cross-validation or test set | -0.0360675 | 0.0611914 | -0.59 | 0.597 | (-0.230806, 0.158671) |
| Definition of pCR | 0.0251448 | 0.2216036 | 0.11 | 0.917 | (-0.6800967, 0.7303864) |
| _cons | 0.8027692 | 0.2599904 | 3.09 | 0.054 | (-0.0246364, 1.630175) |

Table S2 Meta-regression (inverse variance weights, n=10).


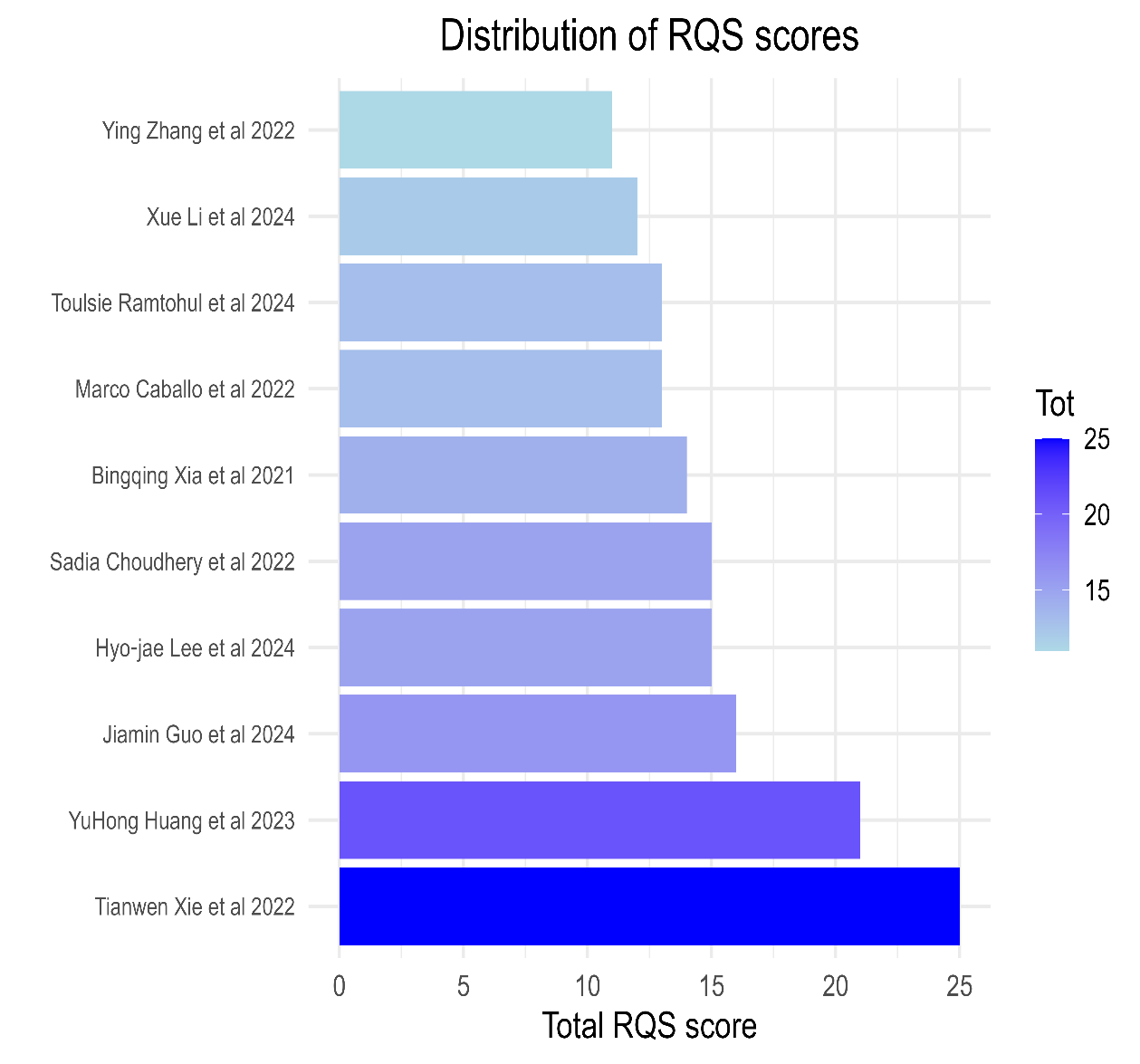


Figer S1 Methodological quality evaluated by using the Radiomics Quality Score (RQS) tool.
